# Maternal, newborn, and children under-five health surveillance system: a scoping review protocol

**DOI:** 10.1186/s13643-023-02378-z

**Published:** 2023-11-22

**Authors:** Asri C. Adisasmita, Trisari Anggondowati, Septyana Choirunisa, Sabarinah Prasetyo, Mardiati Nadjib, Mondastri Korib Sudaryo

**Affiliations:** 1 Department of Epidemiology, Faculty of Public Health, Universitas Indonesia, Jawa Barat, Indonesia; 2 Faculty of Public Health Alumni, Universitas Indonesia, Depok, West Java Indonesia; 3 Department of Biostatistics and Population Studies, Faculty of Public Health, Universitas Indonesia, Jawa Barat, Indonesia; 4 Department of Health Policy and Administration, Faculty of Public Health, Universitas Indonesia, Jawa Barat, Indonesia

**Keywords:** Surveillance, Maternal, Newborn, Children under five, COVID-19, Pandemic

## Abstract

**Background:**

Public health surveillance is crucial in monitoring the progress of maternal, newborn, and children under-five health outcomes (MNCH). Consequently, mapping the existing surveillance system from countries with different income and development levels is needed to learn and compare the effectiveness of surveillance. However, the current COVID-19 pandemic has disrupted the health system, including the healthcare services for pregnant women, neonates, infants, and children under five, as well as the recording, reporting, and surveillance system. The need to adapt to the new normal during the pandemic has stimulated innovation while incorporating new COVID-19-related indicators into the existing public health system. Therefore, this review aims to describe the existing implementation and the COVID-19 pandemic’s influence on the MNCH surveillance system.

**Methods:**

We will search published literature (from MEDLINE, Embase, and *Portal Garuda*), manually search from all reference lists of included studies, and conduct a targeted search of relevant gray literature. This review will include studies of surveillance systems or describe COVID-19 surveillance or routine reports involving MNCH (morbidity and mortality). The studies included will be in English or Indonesian language, observational study designs, and published or documented from 2010 to 2023. Two investigators will independently screen the title and abstract, including each full article to determine the eligibility of studies. The data will be assessed using a narrative approach. Data will be reported in simple descriptive tables.

**Discussion:**

Our findings are expected to map the existing implementation of MNCH surveillance systems before and during the pandemic, including the influence of the COVID-19 pandemic on MNCH surveillance across countries with different income levels. This may contribute to existing knowledge on the MNCH health surveillance system that could be integrated into the surveillance of emerging diseases, such as COVID-19.

**Systematic review registration:**

The protocol has been registered on the Open Science Framework (https://osf.io/bc6t4).

**Supplementary Information:**

The online version contains supplementary material available at 10.1186/s13643-023-02378-z.

## Background

The sustainable development goal (SDG) agenda prioritizes maternal mortality reduction under Goal 3, “Ensure healthy lives and promotes well-being for all at all ages,” targeting to reduce the global maternal mortality ratio to less than 70 per 100,000 live births by 2030 [1, 2]. The progress in achieving the target of maternal, newborn, and children under-five health outcomes (MNCH) must be monitored. Indicators are needed to monitor the progress, including reporting and recording of the indicators and the progress. Indicators might include antenatal care coverage, skilled birth attendance, postnatal care for mothers and newborns, and immunization for children under five.

Thus, the role of public health surveillance becomes imperative. While current and systematic activities of data collection, analysis, and interpretation were aimed to be translated into action [3], surveillance can monitor progress and show which areas need improvement in health programs/policies. Surveillance should facilitate effective program planning and implementation by providing timely and meaningful evidence.

Existing MNCH indicators might vary in definition, data collection method, and the barriers and facilitators in data (indicators) gathering, including its recording and reporting across different regions. The existing reporting and recording, including the MNCH surveillance system of countries with different levels of income and development, must be mapped to learn and compare the effectiveness of surveillance in preventing adverse events across different settings.

The current COVID-19 pandemic has disrupted the global health systems, including healthcare services for pregnant women, neonates, infants, and children under five. A survey conducted by WHO, which involves 105 countries, shows that 53% of the participating countries reported partial disruptions in antenatal care (ANC) and 32% in facility-based deliveries during the early months of the COVID-19 pandemic [4]. More than 60% of 105 countries reported at least partial disruptions in routine immunization [4]. In the Lancet, 118 countries reported several estimated scenarios of coverage reductions of essential maternal and child health interventions, including the prevalence of wasting over 6 months that would represent a 9.8–44.7% increase in under-five child deaths per month and 8.3–38.6% increase in maternal deaths per month [5]. Disruptions in service utilization disproportionately impact those in low to middle-income countries (LMICs) with difficult access to care even before the COVID-19 pandemic [6]. The disruption in MNCH services will affect the recording, reporting, and surveillance systems, hampering routine data collection (locking down health facilities in many areas, many health personnel being infected with COVID-19, etc.). A burden was also added because resources were shifted for pandemic control.

Despite the challenging situation, the health sector must also implement the surveillance of COVID-19 cases and its related indicators. Meanwhile, the need to adapt to a new normal during the pandemic has stimulated innovation while incorporating new COVID-19-related indicators into the existing public health system. Nevertheless, there is a lack of documentation on the effect of the pandemic on MNCH surveillance, so innovations emerged. This scoping review aims to describe (1) the existing implementation of the MNCH surveillance system and (2) how COVID-19 affects the implementation of MNCH surveillance. This scoping review is part of a larger collaborative work that aims to provide evidence to improve the public health surveillance system in Indonesia while expecting it to be applicable to other developing countries as well.

## Methods

The review methods modify the methods used by Arksey and O'Malley (2005) and Kazi MR et al. [7, 8] to systematically review the landscape of the subject matter. According to this framework, there are six different stages in undertaking a scoping review: (1) framing a research question for review, (2) identifying related works to the research question, (3) studying selection, (4) extracting information from the selected articles, (5) summarizing evidence from the identified works, and (6) interpreting and presenting the findings/evidence. This review follows the Preferred Reporting Items for Systematic Reviews and Meta-Analyses extension for scoping reviews (PRISMA-ScR) checklist [9] found in Additional file 1. This review protocol has been registered on the Open Science Framework (https://osf.io/bc6t4).

### Stage 1: Framing a research question for review

Our research questions were developed through consultations with the research (small group) and parent research teams since our study was developed as part of the parent research. The parent research is part of the Partnership in Research Indonesia and Melbourne, which aimed to develop and test a community-based surveillance system on MNCH and other diseases, including COVID-19. This review is expected to contribute to the parent research by enhancing our understanding of the existing MNCH surveillance system and the adaptations/changes occurring during the COVID-19 pandemic. The parent research team comprises academics (epidemiologists (clinical and non-clinical), qualitative researchers, biostatisticians, and health economists) who collaborate with policymakers at the district level (from health authorities and primary health care). We propose to map and synthesize the available literature to describe: (1) what and how is the existing implementation of the MNCH surveillance system before and during the pandemic and (2) how does COVID-19 affect the implementation of MNCH surveillance. We seek information on surveillance conducted at the community and facility-based levels. Table 1 below illustrates the population, concept, and context of this scoping review.

**Table 1:** PCC (Population, Concept, Context) criteria for defining the eligibility of the identified studies

Component	Characteristics
P (Population)	Maternal (pregnancy until postpartum period 6 weeks after delivery), newborn (including perinatal), and children under 5 years
C (Concept)	Implementation of surveillance system development. A routine reporting system will be included if it meets the surveillance system criteria or has the potential to be enhanced into a surveillance system
C (Context)	Articles (quantitative observational studies or qualitative studies) or reports, written in English or Indonesian language and published or documented since 2010, will be included if they describe surveillance in normal situations (before the pandemic) or during the COVID-19 pandemic. There will be no geographical restriction

### Stage 2: Identifying related works to the research questions

#### Search strategy and information sources

A systematic literature search will be conducted by searching electronic databases of the published literature, such as MEDLINE, Embase, and Google Scholar for articles published in the English language, and also an Indonesian-based website namely *Portal Garuda*. The latter is a database of scholarly publications in Indonesia managed by the Ministry of Education, Culture, Research, and Technology. The planned search strategy is shown in Additional file 2. We will also conduct a manual search of additional relevant studies from reference lists of the included studies. To ensure a more thorough review, we will search gray literature from targeted sources, such as relevant government and non-government agencies.

The search strategy is developed by authors and an experienced research librarian. To ensure impartiality, authors and institutions will be blinded. Searches will combine terms capturing two themes, i.e., MNCH surveillance (including morbidity and mortality) and the influence of COVID-19 on surveillance (morbidity, co-morbidity, mortality, vaccine, etc.). This review will only include published articles between 2010 and 2023 that use the English and Indonesian languages; we will exclude languages other than the English and Indonesian languages during article screening and then report how many studies are excluded based on the language restriction (Table 2).

**Table 2:** Search terms for electronic databases

	**Keywords**	**Search terms (English) – MEDLINE and Embase**	**Search terms (in Indonesian language) – *Portal Garuda***
P (Population)	Maternal, newborn, perinatal, infant, children under five	Mother(s), maternal, pregnancy, pregnant, postpartum, neonatal, neonate, newborn, perinatal, infant, baby, children under five	Ibu, maternal, kehamilan, hamil, pasca salin, pasca persalinan, nifas, neonatal, neonatus, bayi baru lahir, bayi, balita
C (Concept)	Surveillance	Surveillance, facility-based surveillance, hospital-based surveillance, facility-based health reporting, community health center surveillance, primary health care surveillance, sentinel surveillance, population surveillance, public health surveillance, community-based surveillance, participatory surveillance, household surveillance, community-based health reporting, routine report, routine data	Surveilans, Surveilans berbasis fasilitas, surveilans berbasis rumah sakit, pelaporan berbasis fasilitas kesehatan, surveilans puskesmas, surveilans sentinel, surveilans populasi, surveilans Kesehatan masyarakat, surveilans berbasis komunitas, pelaporan kesehatan berbasis komunitas, data rutin, surveilans Kesehatan
C (Context):	Situation before the pandemic (no specific keyword is needed) and during the COVID-19 pandemic	COVID, COVID-19, coronavirus, SARS COV-2, sars-coronavirus, corona, vaccine COVID, pandemic	COVID, COVID-19, coronavirus, SARS COV-2, sars-coronavirus, corona, vaksin covid, pandemi

### Stage 3: Study selection

Relevant studies will be screened in two stages: (1) title and abstract review and (2) full-text review. Two out of three reviewers (TA, SC) will screen the title and abstract of all identified articles independently based on the inclusion criteria. Any articles deemed relevant by any of the two reviewers will be included in the next stage of screening, i.e., full-text review. The two reviewers will then independently assess each of the retrieved full texts to determine whether they meet the inclusion criteria. Any disagreement following a full-text screening will be reviewed by the third reviewer (AA) and further discussed among the reviewers until a consensus is reached.

Studies will be included if they describe a surveillance system involving maternal, newborn, infant, and children under 5 years of health (morbidity and mortality) or COVID-19 surveillance or routine reports involving maternal, newborn, infant, and children under five population. Routine reports that meet the surveillance criteria system or reports that could potentially be developed as a surveillance system will be included. The scope of surveillance could be community or facility-based. Studies included can be on any of the following: (a) implementation and (b) development of a new surveillance system responding to COVID-19. Observational study designs, quantitative or qualitative studies, will be included. A systematic review, scoping review, or rapid review will be excluded, but we will review the references in these for inclusion, if applicable.

### Stage 4: Data collection (extracting information from the selected articles)

The research team will develop a data collection instrument to extract study characteristics. We will conduct dual data abstraction, with two reviewers independently extracting data from each included study. To improve accuracy, abstracted data from reviewers will be compared, and any discrepancies will be further discussed to yield consistency between them.

The extracted data will include the following information:

- Author and year of publication
- Study title
- Aim/objective of surveillance
- Setting (country, etc.)
- Type (facility/community/ combination, etc.)
- Data collection and reporting process in the surveillance system (including indicators measured in the surveillance, i.e., coverage of services, such as a skilled birth attendant, coverage of immunization, number of maternal deaths, etc.)
- Measures to improve validity and data quality
- The role of community members or community health workers in the surveillance
- Barriers and facilitators to successful surveillance (including perceptions and experiences of those who were involved in the process)
- Follow-up action (e.g., investigation and review of maternal, newborn, and child deaths morbidity and mortality)
- Results/output or outcomes (including the number of maternal, newborn, and child morbidity and mortality)
- Changes in implementing a surveillance system after the COVID-19 pandemic (e.g., compare completion rate of the indicators and coverage of services between pre- and-post-pandemic).
- Changes in surveillance system (e.g., incorporation of COVID-19 relating indicator to the existing system, implementation on the existing system)

### Stage 5: Summarizing and synthesis of evidence

We will conduct a descriptive analysis of the included studies. Data will be reported in simple descriptive tables and, if possible, will be charted in illustrative graphs or figures. Sub-group analysis will also be reported, for example, by income level of a country (high, middle, and low) and region. No meta-analyses will be performed, and the data will be assessed using a narrative approach.

For our scoping review, the synthesis of evidence aims to describe the implementation of the surveillance system involving the MNCH population. Furthermore, whether the presence of the COVID-19 pandemic alters the existing surveillance system. The findings of this review will be described in comparison between developed and developing countries to allow sharing of lessons learned and experiences.

### Stage 6: Interpret and present the findings/evidence

All the information extracted will be described according to the research questions. For research question number 1, the findings will be presented based on the following topics: (a) aim/objective, (b) methods, and (c) output of the surveillance implementation. For research question number 2, the findings on the influence of the pandemic will be presented based on (a) incorporation of the COVID-19-related indicator to the existing system and (b) implementation on the existing system (negative influence, such as disruption, and positive influence, such as modification/alteration of data gathering using digital data).

Furthermore, we will identify and present gaps in the research area to give readers an understanding of the landscape of surveillance implementation for MNCH before and during the COVID-19 pandemic, including what future action must be taken. Findings from this scoping review might serve as a foundation for further potential studies and systematic reviews.

## Discussion

Since a scoping review can map the concepts underpinning a research area and the main sources and types of available evidence, the aggregated findings provide an overview of the research rather than an assessment of the quality of individual studies. Our scoping review will provide important insights for policymakers in different settings. The methods of this proposed scoping review identify and map the existing health surveillance system and routine reports, which could be upgraded into surveillance that includes a comprehensive and well-defined approach, as listed to such a degree to allow for replication. This scoping review aims to map (1) the existing implementation of the MNCH surveillance system before and during the pandemic and (2) the influence of the COVID-19 pandemic on MNCH surveillance across countries with different income levels. The findings of this scoping review will add to the existing knowledge on the MNCH health surveillance system that could be integrated into the surveillance of emerging diseases, such as COVID-19. Furthermore, the MNCH surveillance system could then be modified so that the influence of COVID-19 on MNCH data could be recorded and integrated. Thus, the burden of mortality among mothers, newborns, and children under five is expected to be reduced while simultaneously serving as a monitoring tool to track progress.

### Supplementary Information

**Additional file 1.** Preferred Reporting Items for Systematic Reviews and Meta-Analyses extension for Scoping Reviews (PRISMA-ScR) Checklist.

**Additional file 2.** Search Strategies.

## Data Availability

Data sharing is not applicable to this protocol.

## Abbreviations

MNCH: Maternal, newborn, and child under-five health
SDGs: Sustainable development goals
ENAP: Every newborn action plan
EPMM: Ending preventable maternal mortality
ANC: Antenatal care
FBD: Facility-based delivery
LMICs: Low- and middle-income countries
MeSH: Medical subject heading
