# Supplementary material for: Maternal, newborn, and children under-five health surveillance system: a scoping review protocol
Source: Syst Rev. 2023 Nov 22;12:220. doi: 10.1186/s13643-023-02378-z (PMC10664649; doi:10.1186/s13643-023-02378-z)
Supplement: Supplementary file 2 — Additional file 2. Search Strategies. [file 13643_2023_2378_MOESM2_ESM.docx]

**Additional File 2**

**Search Strategy (draft)**

**EBSCO-Medline (before pandemic)**

1. Mothers (MeSH)
2. Mother (TI, AB)
3. 1 OR 2
4. Infant, Newborn (MeSH)
5. EXPLODE Infant
6. Newborn OR neonate OR infant OR baby OR perinatal OR maternal (TI, AB)
7. 3 OR 4 OR 5 OR 6
8. “children under 5” OR “children under 5 years” OR “children under five” OR “children under five years” (TI, AB)
9. Child, Preschool (MeSH)
10. 7 OR 8 OR 9
11. Pregnancy (MeSH)
12. Pregnan* (TI, AB)
13. Postpartum (TI, AB)
14. 10 OR 11 OR 12 OR 13
15. “Public Health Surveillance” (MeSH)
16. surveillance OR "facility-based surveillance" OR "hospital-based surveillance" OR "facility-based health reporting" OR "community health center surveillance" OR "primary health care surveillance" OR "sentinel surveillance" OR "population surveillance" OR "public health surveillance" OR "community-based surveillance" OR "participatory surveillance" OR "household surveillance" OR "community based health reporting" OR "community based health reporting" OR "routine report" OR "routine data" (TI) OR reporting OR monitoring OR tracking OR data collection OR indicators
17. “Sentinel Surveillance” (MeSH)
18. 13 OR 14 OR 15
19. 12 AND 16
20. “COVID-1” (MeSH) OR “COVID19 Vaccines” (MeSH) OR “2019-nCoV Vaccine mRNA-1273” OR “ChAdOx1 nCoV-19”
21. covid-19 OR coronavirus OR 2019ncov OR sars-cov-2 OR cov-19 OR pandemic OR "2019 novel coronavirus" OR "coronavirus disease" (TI, AB)
22. 18 OR 19
23. 17 NOT 20
24. Limit to 2010-2023
25. Limit to full-text
26. Limit to human

**EBSCO-Medline (pandemic)**

1. Mothers (MeSH)
2. Mother (TI, AB)
3. 1 OR 2
4. Infant, Newborn (MeSH)
5. EXPLODE Infant
6. Newborn OR neonate OR infant OR baby OR perinatal OR maternal (TI, AB)
7. 3 OR 4 OR 5 OR 6
8. “children under 5” OR “children under 5 years” OR “children under five” OR “children under five years” (TI, AB)
9. Child, Preschool (MeSH)
10. 7 OR 8 OR 9
11. Pregnancy (MeSH)
12. Pregnan* (TI, AB)
13. Postpartum (TI, AB)
14. 10 OR 11 OR 12 OR 13
15. “Public Health Surveillance” (MeSH)
16. surveillance OR "facility-based surveillance" OR "hospital-based surveillance" OR "facility-based health reporting" OR "community health center surveillance" OR "primary health care surveillance" OR "sentinel surveillance" OR "population surveillance" OR "public health surveillance" OR "community-based surveillance" OR "participatory surveillance" OR "household surveillance" OR "community based health reporting" OR "community based health reporting" OR "routine report" OR "routine data" (TI) OR reporting OR monitoring OR tracking OR data collection OR indicators
17. “Sentinel Surveillance” (MeSH)
18. 13 OR 14 OR 15
19. 12 AND 16
20. “COVID-1” (MeSH) OR “COVID19 Vaccines” (MeSH) OR “2019-nCoV Vaccine mRNA-1273” OR “ChAdOx1 nCoV-19”
21. covid-19 OR coronavirus OR 2019ncov OR sars-cov-2 OR cov-19 OR pandemic OR "2019 novel coronavirus" OR "coronavirus disease" (TI, AB)
22. 18 OR 19
23. 17 AND 20
24. Limit to 2010-2023
25. Limit to full-text
26. Limit to human
